# Design of a portable machine for picking chamomile flowers

**DOI:** 10.1038/s41598-026-39880-y

**Published:** 2026-03-11

**Authors:** M. A. M. Abd El-Moulaa, Ashraf K. Zaalouk, Wael A. Mahmoud

**Affiliations:** 1 Agricultural Machinery and Power Engineering Department, Faculty of Agricultural Engineering (Assiut Branch), Al-Azhar University, Assiut, Egypt; 2 Agricultural Machinery and Power Engineering Department, Faculty of Agricultural Engineering, Al-Azhar University, Cairo, Egypt

**Keywords:** Chamomile, Harvesting, Machine, Productivity, Mechanization, Engineering, Plant sciences

## Abstract

This research details the design, fabrication, and performance evaluation of a novel, portable machine for mechanized harvesting of chamomile flowers. The machine incorporates a specialized picking mechanism with a variable-length comb, a reciprocating cutting blade, and a collecting brush, powered by independent lithium-ion battery units for field portability. A systematic experimental investigation was conducted to determine the effects of key operational parameters on productivity, including comb length, comb gap width, and cutting blade speed. The study identified optimal conditions that yielded the highest productivity of 31.74 kg/h. This was achieved with a 100 mm comb length, a 5 mm comb gap, a 5.15 m/min blade speed, and a 200 rpm brush rotation. These findings provide crucial insights for the optimal design and operational parameters of chamomile harvesting machinery, facilitating efficient mechanization.

## Introduction

German chamomile (*Matricaria chamomilla L.*), an annual plant in the Asteraceae family, is one of the world’s most widely used herbs. Its small flowers, with white petals surrounding a bright yellow center, are a familiar sight, as the herb grows wild in many parts of the world and is also cultivated for use in dried teas, pharmaceutical products, and the production of essential oil. Chamomile flowers contain blue essential oil in concentrations ranging from 0.2 to 1.9%^1^. This essential oil contains azulene, used in perfumery, cosmetic creams, hair preparations, skin lotions, toothpaste, and fine liquors. Chamomile is considered beneficial in medicinal applications. The use of nano-encapsulated chamomile emulsion resulted in a significant reduction in breast tumor incidence, restoration of oxidative stress response, enhanced immune response, and reduced inflammation^2^. Chamomile flowers appear in flushes, with 4–5 flushes typically obtained. The importance of medicinal aromatic plants lies in their being a rich source of natural active substances such as alkaloids, flavonoids, terpenes, and others, which can be extracted Ibrahim^3^. The second, third, and fourth flushes are the most significant contributors to flower yield. Flowering is so profuse that, practically every alternate day, at least 30–40 labor units are required to pluck flowers from a 0.25–0.3 ha area^4^. Manual picking yields low amounts, typically around 3–5 kg/h^5^. When designing any mechanical machine, understanding the physical properties of the materials the machine will handle or be made from is crucial. These properties are the foundation for determining the machine’s efficiency and performance. Elkaoud et al.^6^. The threshing process plays a crucial role in assessing product quality, and the utilization of threshing equipment offers significant benefits in terms of enhanced product quality, reduced processing time, and minimized labor requirements. Ali et al.^7^ Given these challenges, mechanization is crucial, as large-scale production can only be realized through mechanical harvesting. Consequently, various chamomile-producing countries, including Argentina, Slovakia, Serbia, Italy, and Germany, currently employ mechanized harvesting techniques based on different picking principles^8^.

Chamomile flower harvesters have undergone significant evolution. Early models simulated manual harvesting, with the drive motor primarily used for combining movement, while inflorescence harvesting occurred through the interaction of harvester motion and a combing procedure^9^. Subsequent generations of chamomile harvesters featured more complex constructions. Beyond locomotion, their drive motors also powered other working organs. These devices were designed as rotors (drums) with combs positioned around their circumference.

Among the various picking principles, linearly moved picking combs without stalk cutting are commonly found in hand-held devices and manually pushed picking carts. These handheld tools can achieve picking amounts of about 10–15 kg of fresh flowers per hour^5^. To minimize residual stalk length, a circulating chain drive with shearing bars has been proposed, or rotating drums equipped with blades mounted above the comb section that run against the comb sections to shear flower heads at the comb base^10^. Another picking method utilizes a wedge-shaped pulley shaft instead of a cutting wire to impact and tear out stems while rotating in the driving direction. This shaft is located below the comb tine. Above the tines, a paddle rotates against the driving direction, guiding flower heads over a knife for separation from their stems^11^. In a third variation, a cutter bar with knives is installed below the comb tines to cut stem rests directly beneath the flower heads^12^.

The LINZ III harvester is a forerunner and foundational machine for both Russian and German designs^13^. Furthermore, the picking unit was engineered to meet dual demands by incorporating double-layer combs arranged on blades within a rotating drum. This comb unit comprises two identical comb layers, functioning akin to a “mini cutter-bar” for grass mowers^14^. The necessity for developing specialized mechanical harvesting mechanisms is evident, as there remains a notable lack of suitable equipment for the harvesting of fine-grain and sensitive crops. These crops, primarily grown in limited acreages, necessitate a specialized approach to ensure the highest efficiency with minimum damage, which justifies the design of small and optimized machines^15^.

This research aims to design and manufacture a machine suitable for small and medium-sized holdings for the mechanization of chamomile flower picking, ensuring excellent picking quality with minimal flower damage, and to systematically investigate the effects of various comb lengths, comb gap widths, cutting blade speeds, and brush rotation speeds on machine productivity and the cost of the flower picking operation.

## Materials and methods

### Engineering design basis

The conceptual design methodology for the portable chamomile picking machine was grounded in precise scientific principles and distinct engineering and operational criteria, aiming to achieve maximum harvesting efficiency while minimizing losses and damage. The determination of the physical, mechanical, and aerodynamic properties of chamomile flowers and their stems served as the cornerstone for defining detailed design specifications. These properties were comprehensively established in a previous study Abdelmoulaa et al.^16^, which provided crucial fundamental data such as average flower diameter, mass, picking force, shear force, static friction coefficients, and terminal velocity. These values were considered key inputs for determining the dimensions and operational specifications of the machine’s components. In addition to this scientific foundation, a set of operational goals guided the design process to ensure the machine meets practical needs, including achieving high picking efficiency, minimizing mechanical damage, ensuring portability and ease of use, suitability for small agricultural holdings, and reliance on a low-voltage electrical power source.

### Picking machine

**Fig. 1:**
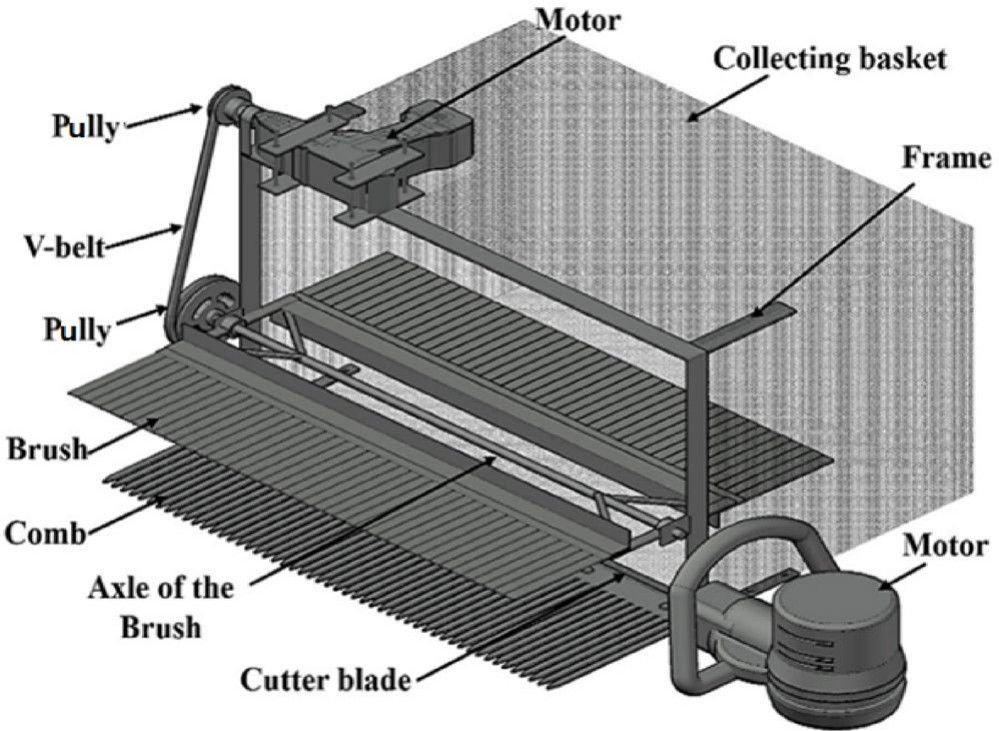
Isometric drawing of the developed machine for chamomile flower picking.

The harvesting machine shown in Fig. 1 consists of the following main components: a frame, picking mechanism, axis, transmission system, collecting basket, and power source. All design dimensions of these components were determined based on the results according to Abd El-Moulaa et al. ensuring its engineering properties were precisely calculated for optimal performance.

#### A- Frame

The picking machine frame is made of a rectangular steel flat bar (2 × 20 mm) and dimensions of 505 mm width, 305 mm height, to assemble the parts of the machine.

#### B- Picking mechanism

The picking mechanism consists of three parts that work together to perform one job, which is picking flowers by fixing the stems of flowers in a vertical position in the direction of movement of the machine, directing the flowers toward the cutting mechanism, and then collecting them in a flower collection box. The picking mechanism consists of the following parts:

1. Comb
  The basic function of the comb is to collect and stabilize plant branches that contain flowers, and to reserve the flowers at the top of the comb to make it easier to cut them from the bottom of the comb. The comb was fabricated from steel Sects. 500 mm in length, 2 mm in thickness, with four variables (lengths of teeth) of 50, 100, 150, and 200 mm (for comparison). Each tooth of the comb has a fixed width of 5 mm, while the distance between the teeth is variable (3, 5, 7, and 9 mm) to test the machine to achieve the best results. The edge of each tooth is beveled in the shape of an isosceles triangle with a side length of 10 mm, to make it easier for the comb to penetrate dense plant branches. All combs are designed by CNC machines to ensure the best operating results. The comb is easy to disassemble and fix, as it is attached directly to the bottom of the frame with four screws. Details of the cutting blade design and the mechanism used to fix it to the comb assembly are illustrated in Fig. 2.

**Fig. 2:**
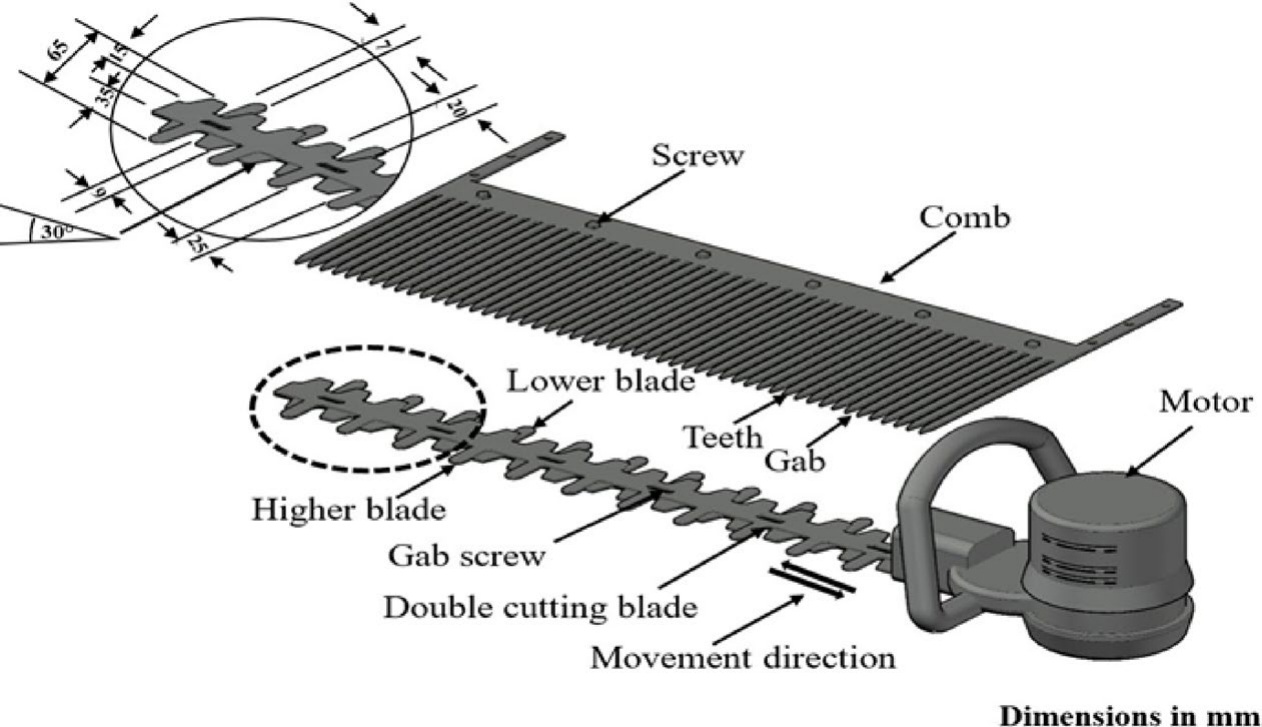
The cutting blade and the method of fixing it on the comb.
2. Cutting blades
  The double cutting blade works to cut the flower stems that are lined up via the teeth of the comb. It is attached to the bottom of the comb using screws that have a longitudinal path of the same size as the length of the reciprocating path (Fig. 3). The cutting mechanism is equipped with two blades that operate with a reciprocating motion. The comb, which collects the flowers, consists of a number of gaps determined by the studied variable, the Comb gap width. The comb is designed so that the number of gaps changes according to the tested gap width to ensure picking efficiency. The two blades operate along the entire working width of the comb, ensuring that the flowers collected by these gaps are effectively sheared.

**Fig. 3:**
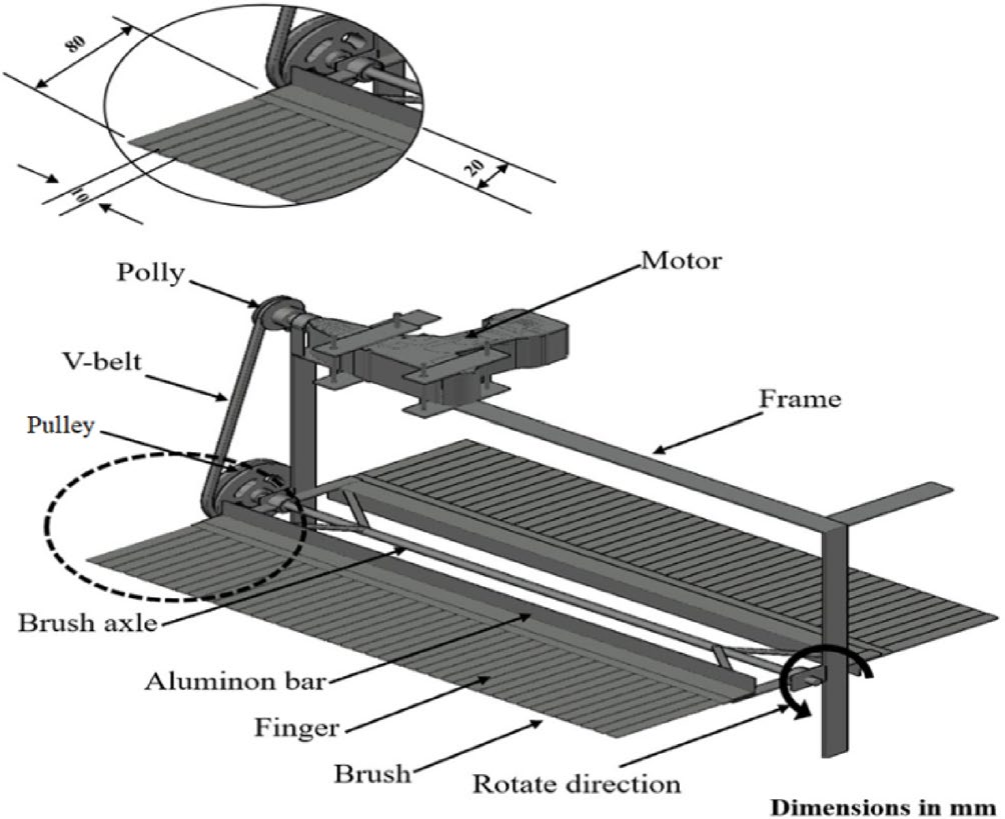
The collecting brush of the picking machine for chamomile flowers.
  The length of the cutting blade is 500 mm, its width is 70 mm, and its thickness is 1 mm. Each blade is beveled at an angle of 30° to ensure obtaining the best cut. All blades are designed by a 3D CNC machine to ensure obtaining the best operating results. The cutting blade moves in a reciprocating motion, using a power take-off from the speed reduction assembly of an electric motor, powered by a 20-V battery. The voltage is controlled by a DC-DC converter to obtain a variable speed of the reciprocating cutting blade.
3. Collecting brush
  The collecting brush performs two functions: it guides the flowers to line up precisely within the comb gaps, and it gently conveys the cut flowers into the collection box. The mechanism utilizes two sets of segmented rubber fingers, facing each other, fixed around a rotational axis (Fig. 3). Each individual finger has dimensions of 100 mm in length, 10 mm in width, and 2 mm in thickness.
  The use of segmented rubber fingers (in lieu of a solid flat rubber blade) was selected based on critical engineering and experimental considerations. The segmented design without noticeable gaps ensures high flexibility, leading to superior ease of rotational movement by reducing the combined resistance from the collected mass. This segmented approach was essential as preliminary trials showed that a solid flat blade caused significant Beater Jamming and difficult rotational movement. Furthermore, this design provides a gentle and localized handling force on the delicate flowers to mitigate mechanical shock and reduce crushing probability, while increasing collection efficiency by preventing the accumulation of the picked mass on a wide surface.

#### C- Axis

The axis of the collecting brush rotates horizontally and is made of steel, with a diameter of 8 mm and 550 mm length, and it is based on a pair of bearings fixed by the frame. The vertical distance from the axle to the comb is 125 mm.

#### D- Transmission systems of power

1. Collecting brush
  The axis of the collecting brush is powered by an electrical motor ( FIT, DR 70 -Dc, 1350 rpm 25 N.m, 12 V, and 2A) and rotates with a pulley (108, 135 and 180 mm), which takes its movement from a V-belt connected to the pulley (65 mm) of the motor shaft. A schematic power transmission system of the picking machine is shown in Fig. 4.

**Fig. 4:**
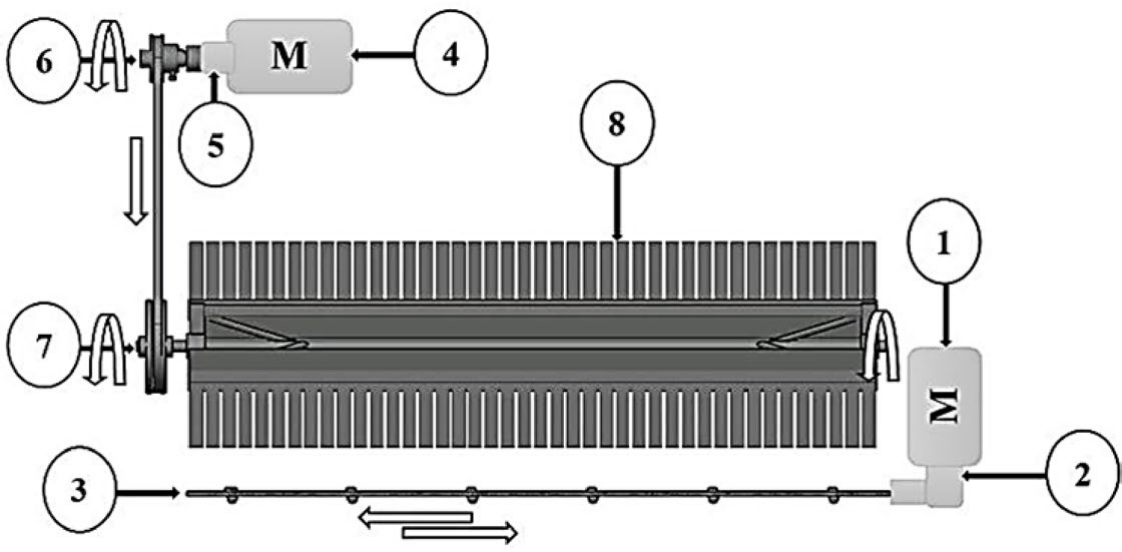
Schematic power transmission system of the picking machine.
2. Cutting blade
  The cutting blades move in a reciprocating motion; the clearance between them makes it work like a pair of scissors. The cutting blade is powered by an electrical motor (TOTAL, P20S -Dc, 2800 rpm, 35 N.m, 20 V and 2A). The motor is attached to an internal gearbox, and the blade is fixed by an arm extending from it. The reciprocating speeds are changed by DC-DC converter to 12, 16, and 20-V. A schematic power transmission system of the picking machine shown in Fig. 4.

#### E- Collecting basket

The collecting basket chassis was made of metal bars and covered with a changeable fabric net bag, holding approximately 5 kg of flowers, which can be emptied very easily.

Where 1, Cutting blade motor; 2, Gearbox; 3, Cutting blade; 4, Collecting brush motor; 5, Gearbox; 6, Pulley (D = 65 mm); 7, Pulley (D = 108, 135, and 180 mm); 8, Collecting brush.

#### E- Power source

1. Collecting brush motor
  The lithium battery is the main source of power for collecting brush motors (FIT, 12 V and 2 Ah).
2. Cutting blade motor
  The lithium battery is the main source of power for cutting blade motor (TOTAL, 20 V and 2 Ah).

### Measuring instruments

Balance: to measure the mass of the flowers (accuracy 0.01 g).

Stopwatch: to record the time consumed during calculation productivity in different experiments.

A tachometer was used to measure rotational speed.

Digital multimeter the power requirement (kW) was determined by using the digital multimeter to measure the line current strength (I) and the potential difference value (V).

### Field experiment and operation procedures

The experimental investigation was conducted in a homogeneous chamomile field cultivated with uniform spacing at the Royal Herbal Company farm during the 2021–2022 agricultural season, in order to provide a reliable testing environment. The test area was divided into experimental units to ensure the separation of variable effects. Each combination of the operational variables was repeated three times to ensure the reliability of the results.

**Fig. 5:**
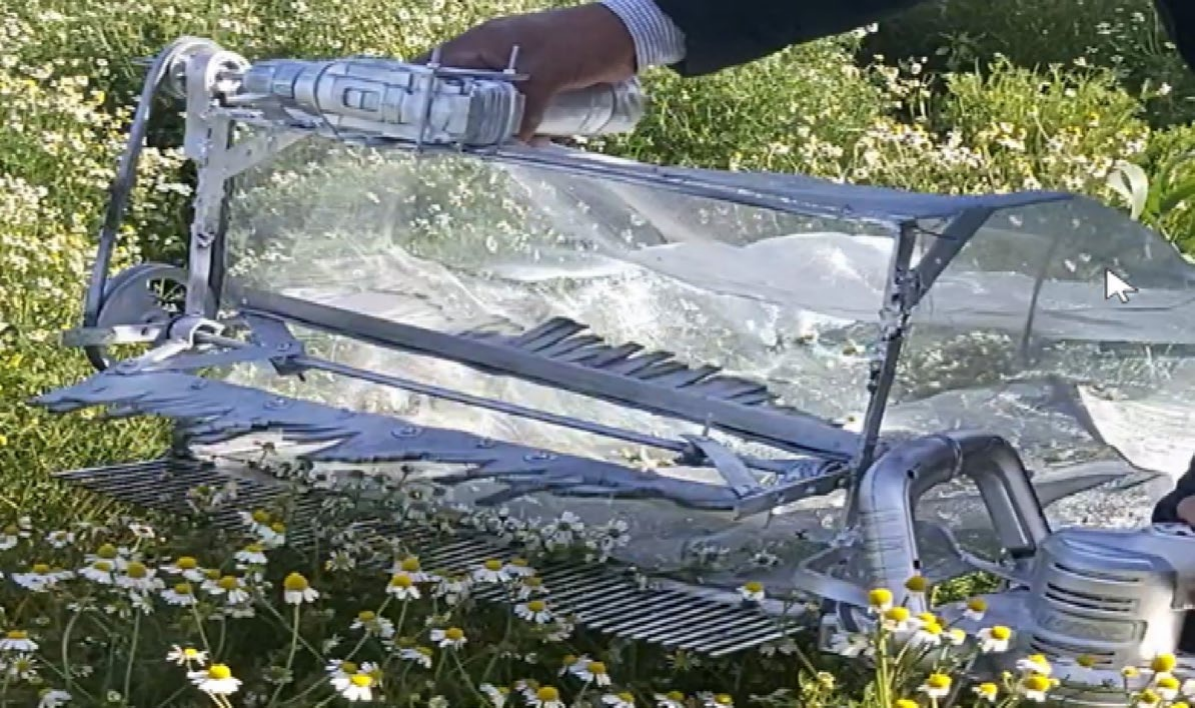
Image of the developed picking machine during operation.

During machine operation, the operator was instructed to follow a predetermined path and maintain a uniform and consistent walking speed as much as possible while passing through each experimental unit. The portable picking machine operation involves using both hands to guide the machine through the chamomile rows. As the machine is pushed toward the rows, the collecting brush’s pushing force assists in guiding the flowers toward the comb and the cutting mechanism. The actual picking time *(T)* for each run was precisely recorded using a stopwatch, and the total mass of picked flowers *(mp)* was weighed using a digital balance immediately after the completion of each experiment. The developed machine during actual field operation, illustrating the guiding process through the chamomile rows, is shown in Fig. 5.

### Experimental variables and evaluation metrics

To assess the performance of the novel chamomile picking machine, a systematic experiment was conducted to study the effect of four key operational variables on the machine’s productivity. These variables were selected based on the design of the picking mechanism and preliminary observations, and included:

1) Picking comb length (tested at 50, 100, 150, and 200 mm).

2) Comb gap width (tested at 3, 5, 7, and 9 mm).

3) Cutting blade speed (tested at 3.01, 4.11, and 5.15 m/min).

4) Collecting brush speed (tested at 150, 200, and 250 rpm).

Each combination of operational variables was repeated three times to ensure reliability. The primary evaluation factor adopted was Machine Productivity *(P*_*m*_*)*, which represents the total mass of picked flowers per unit of time. Machine productivity *(P*_*m*_*)* was determined using the following equation according to^17^ and^18^and ^19^:

$$P_{m} = \, m_{p} /T$$

where:

P_m_: Machine productivity (kg/h).

m_p_: Total mass of the picking flowers (kg).

T: Picking time (h).

### Field experiment procedures and data collection

To execute the experimental investigation, the homogeneous chamomile field at the Royal Herbal Company’s farm was divided into experimental units to ensure the separation of variable effects.

The following operational and measurement protocol was adhered to in order to ensure the accuracy of the data used in calculating the machine’s productivity (*P*_*m*_):

- *Operation and Time Protocol (T)* Operators were instructed to maintain a uniform and consistent walking speed as much as possible while passing through each experimental unit. The actual picking time *(T)* for each run was precisely recorded using a stopwatch.
- *Mass Measurement Protocol* (*P*_*m*_) Immediately after the machine completed its run for each replicate, the picked flowers were emptied from the collecting basket. The total mass of the picked flowers (*m*_*p*_) was weighed using a digital balance.
- *Data Processing* The collected results were processed by calculating the arithmetic mean of the results from the three replicates for each operational variable, and these averages were used in calculating the final productivity (*P*_*m*_).

## Results and discussions

The performance of the prototype was systematically evaluated to determine the effects of the four main operational factors: comb length, comb gap width, cutting blade speed, and brush rotation speed, on system productivity. The subsequent Analysis of Variance (ANOVA) confirms that all these factors have a highly statistically significant effect on the machine’s performance, providing a robust statistical foundation for the conclusions drawn. The results, illustrated primarily in Fig. 7, reveal general trends where productivity consistently improves under optimal settings, which will be further discussed in terms of their individual and interactive effects.

### Analysis of variance (ANOVA)

Operational parameters on machine productivity were analyzed using a multi-factor Analysis of Variance (ANOVA). Table 1 clearly demonstrates that all four tested main factors, Comb Length (l), Comb Gap Width (g), Brush Rotation Speed (w), and Cutting Blade Speed (v), had a highly statistically significant effect on the machine’s productivity (P < 0.0001). This high level of significance confirms that the variation in productivity is reliably attributable to these variables, rather than experimental chance.

**Table 1:** Analysis of variance (ANOVA) results for the effect of brush rotation speed, cutting blade speed, comb gap width, and comb length on chamomile harvesting machine productivity.

*P*-value	F-ratio	MS (mean square)	SS (sum of squares)	DF (degrees of freedom)	Source
0.0000	2198.85	627.44	1254.87	2	Brush speed (w)
0.0000	1049.06	299.35	598.70	2	Cutting blade speed (v)
0.0000	2392.02	682.56	2047.67	3	Comb gap width (g)
0.0000	3794.26	1082.68	3248.04	3	Comb length (l)
0.0000	68.15	19.45	77.78	4	w*v
0.0000	18.31	5.23	31.35	6	w*g
0.0000	29.86	8.52	51.13	6	w*l
0.0000	16.81	4.80	28.79	6	v*g
0.0000	17.32	4.94	29.66	6	v*l
0.0000	249.79	71.28	641.48	9	g*l
0.4700	1.002	0.2858	13.43	47	Residual interactions
		0.2853	82.17	288	Error (residual)
			8095.84	431	Total

Upon examining the F-Ratio values to ascertain the relative contribution of these factors, it was determined that Comb Length (l) is the most dominant factor influencing machine productivity (F = 3794.26), followed closely by Comb Gap Width (g) (F = 2392.02). This strong statistical evidence underscores the primary role of the geometric dimensions of the picking mechanism in determining the potential yield. Crucially, all two-way interactions between the variables also showed a highly significant statistical effect, necessitating that the subsequent discussion analyzes each factor not in isolation, but as part of an integrated, interacting operational system.

### Effect of cutting blade speed on productivity

**Fig. 6:**
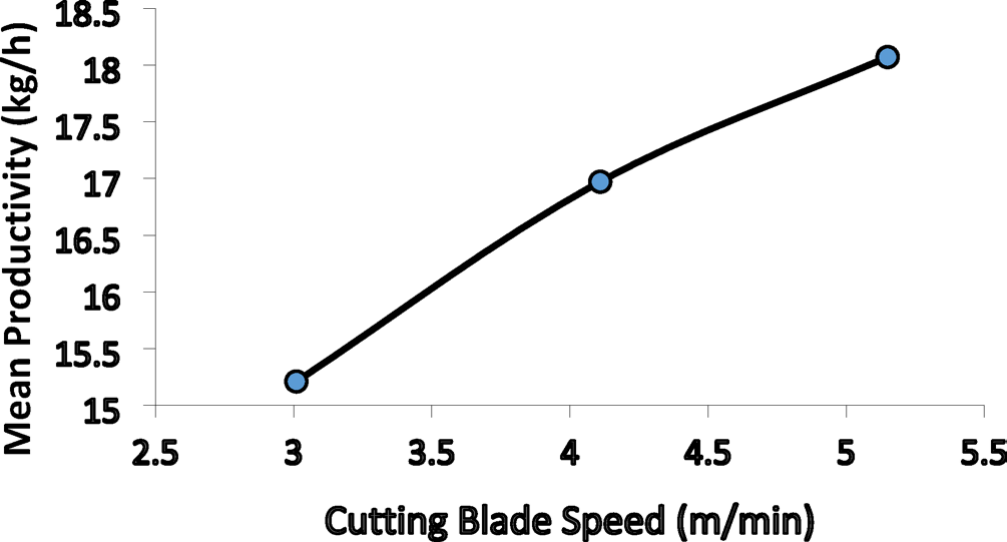
The effect of cutting blade speed on the mean productivity of the chamomile harvesting machine.

**Fig. 7:**
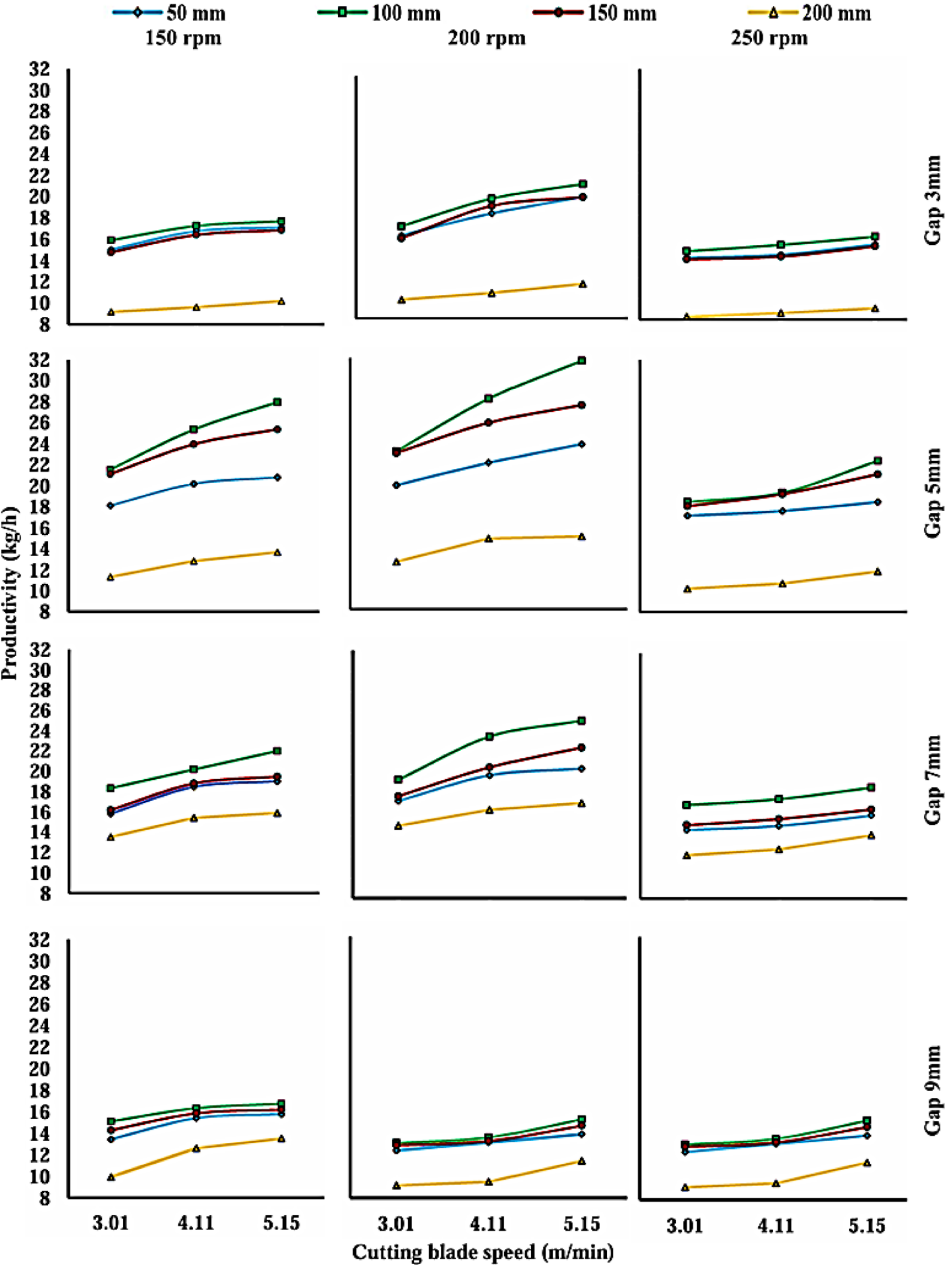
Effect of cutting blade speed on productivity at four different levels of comb length, four different levels of the comb gap width, and three rotation speeds of the brush.

Based on the results shown in Fig. 6, and the ANOVA, the cutting blade speed (v) was confirmed to have a highly statistically significant effect on machine productivity (Fig. 7). The curve clearly indicates that productivity increases continuously with the increase in speed, where the highest mean productivity (18.070 kg/h) was recorded at the maximum tested speed (5.15 m/min).

This positive trend is rooted in the mechanical principles of the reciprocating cutting process. The increase in the blade’s linear speed directly leads to a reduction in the Flower-Blade Interaction Time. The shorter this time, the higher the successful shearing rates and the lower the probability of undesirable phenomena. High speed effectively and rapidly applies the Required Shear Force to the flower stem, preventing Stem Deflection or Jamming within the comb gaps. Consequently, losses are reduced, and the number of successfully cut flowers per unit time increases, explaining the peak productivity at the maximum blade speed and confirming the necessity of operating at the highest possible blade speed.

Although the marginal mean analysis indicated a peak productivity of 18.070 kg/h for cutting speed, the highest overall productivity achieved in the experiment was 21.31 kg/h. This optimal result was realized under the specific interactive combination of the highest levels for all four factors (3 mm comb gap, 100 mm comb length, 5.15 m/min cutting blade speed, and 200-rpm brush rotation speed.

### Analysis of brush speed performance

At 150 rpm: This is considered a low speed, and the brush does not rotate fast enough to effectively collect all the cut flowers and drop them into the collection hopper. This results in product loss, as some flowers may fall to the ground, reducing overall productivity.

At 200 rpm (optimum): This speed strikes the perfect balance for efficient collection. It is fast enough to collect all the cut flowers without damaging the delicate flower heads or creating turbulent airflow. This speed ensures the maximum amount of harvested material is successfully collected, resulting in peak productivity.

250 rpm (very fast): When the brush speed is too high, it creates a strong sweep and more intense airflow. This can cause some of the uncut flowers to be thrown in front of the comb, resulting in material loss and reduced productivity.

### Effect of comb gap width on productivity:

A detailed examination of productivity trends across different comb gap widths was conducted. The results showed that the 5 mm gap consistently yielded the highest overall productivity.

- 5 mm Gap: The maximum productivity reached 31.74 kg/h, which was achieved under the optimal conditions: a 100 mm comb length, a 5.15 m/min blade speed, and a 200-rpm brush rotation speed.
- 7 mm Gap: The maximum productivity was slightly lower at 25.19 kg/h, achieved under the same optimal conditions (100 mm comb length, 5.15 m/min blade speed, and 200 rpm brush rotation speed).
- 9 mm Gap: The maximum productivity was 19.78 kg/h, and its value was consistently lower compared to the 5 mm and 7 mm gaps.

Based on the data obtained, it was observed that a narrow gap (3 mm) leads to flow restriction of the chamomile stems, causing significant dynamic resistance. This resistance can impede the machine’s movement and negatively affect its maneuverability, potentially causing flowers to be pushed aside instead of being picked. In contrast, when the gap is too wide (7 mm and 9 mm), the picking mechanism loses its capture efficiency. Many flowers pass through the gap without being properly cut, leading to a noticeable drop in harvesting productivity.

Therefore, it is evident that the 5 mm gap width achieved an ideal balance. It is wide enough to ensure a smooth flow of plants and prevent blockages yet narrow enough to effectively capture the stems and present them to the cutting blade. This width is perfectly suited to the average diameter of the chamomile stems, which explains its high operational efficiency.

### Effect of comb length on productivity

The data regarding the effect of comb length on productivity showed a clear pattern: productivity generally increases when the comb length is extended from 50 to 100 mm, but consistently decreases with further increases to 150 mm and 200 mm.

- At a brush rotation speed of 150 rpm and a 3 mm comb gap, productivity increased when the comb length went from 50 to 100 mm (from 15.04 to 15.91 kg/h at a 3.01 m/min blade speed). In contrast, it sharply decreased when the length was increased from 150 to 200 mm (from 14.77 to 9.17 kg/h at the same blade speed).
- This pattern was replicated across all tested brush rotation speeds and comb gap widths.

Based on the test results, it can be interpreted that the 100 mm comb length is the most optimal from an engineering perspective for the picking mechanism. This length achieves an ideal balance between the efficiency of flower collection and the reduction of resistance faced by the machine. While the 100 mm length provides a sufficient surface area to collect many flowers, it prevents the entanglement of stems and motion obstruction that can occur with the longer comb (200 mm). A shorter comb (150 mm) may not be able to pick enough flowers. Additionally, this length provides the operator with better control over the machine, allowing for precise guidance through the plants without causing damage, thereby increasing harvesting efficiency.

In essence, these findings underscore the intricate interplay between cutting blade speed, brush rotation speed, and comb gap width. The data consistently shows that the 5 mm comb gap, in conjunction with the 100 mm comb length and the identified optimal parameters (5.15 m/min cutting blade speed and 200 rpm brush rotation speed), represents the most efficient configuration for maximizing the system’s output. These results provide critical insights for the design and operational optimization of similar processing systems, emphasizing the need for precise parameter tuning to achieve maximum output.

### Analysis of performance under varying operational and seasonal conditions

In the context of this preliminary study of the machine’s performance, the analysis extends to include observations regarding varying operational and seasonal conditions that were not subjected to systematic analysis as controlled variables. The experiments were conducted during the agricultural season, considering that chamomile flowers appear in multiple flushes, with the second, third, and fourth flushes contributing most to the overall flower yield. The best productivity results recorded (31.74 kg/h) were associated with operating during the period of these main flushes. It must be noted that changes in the flowering stage (from one flush to another) can affect the durability and physical properties of the flower, representing a seasonal factor of variation that warrants further research in the future. Furthermore, given that the machine is designed to be operated by a single person, any variability in the operator’s walking speed in the field can be considered a case study for different loading conditions that directly affect the picking time *(T)* and, consequently, the machine’s productivity (*P*_*m*_), thus emphasizing the importance of training operators to ensure optimal efficiency.

## Conclusion

The findings of this study confirm the successful design and fabrication of a portable machine for harvesting chamomile flowers. The research identified the optimal operational parameters for maximizing productivity. The key conclusions are as follows:

- The machine’s design successfully addresses the need for a portable and efficient solution for chamomile harvesting.
- A cutting blade speed of 5.15 m/min and a brush rotation speed of 200 rpm were found to be optimal for achieving maximum productivity.
- A comb length of 100 mm and a comb gap width of 5 mm are the most effective configurations for maximizing flower collection.
- The study provides a clear set of optimal conditions that can be applied to enhance the performance and efficiency of future chamomile harvesting technologies.

Essential to this conclusion is the understanding that the results obtained from the analysis of variable effects in this study represent preliminary trends for the prototype’s performance, and should be viewed as design guidelines requiring further evaluation and verification under various and extensive field conditions before being generalized as conclusions.

## Data Availability

All data generated or analyzed during this study are included in this published article.
